# Annual Meeting of the Chicago College of Pharmacy

**Published:** 1868-04

**Authors:** 


### ANNUAL MEETING OF THE CHICAGO COLLEGE OF PHARMACY.

A meeting of the members of the Chicago College of Pharmacy was held March 10th, in their new rooms in Rice's building. The meeting was largely attended by the druggists and physicians of the city. E. H. Sargent occupied the Chair. The Secretary called the roll, and read the minutes of the last meeting.

### REPORTS OF COMMITTEES.

The different Committees then reported. H. D. Garrison, on Apparatus, reported that many additions had been made to that of the College, but he did not know the exact condition of apparatus at this time.

Mr. Mills, on the Library Committee, reported that the Library fund amounted to $725, $500 of which had been appropriated by the Board of Trustees, to be expended in the purchase of books. The Committee suggested that the remaining portion of the fund be appropriated at this meeting for the same purpose.

The Committee on Lectures, reported that two lecturers could easily be obtained, and the matter had been referred to the Board of Trustees.

The Secretary and Treasurer's reports were then presented. The latter reported $1,500 in the Treasury Sept. 1, and gave the amounts that had been expended, which nearly covered the receipts.

Upon motion, the Special Committee on Lectures was discharged.

The Committee on Examination reported the names of several persons who had applied for membership. As several of the applications were without the necessary indorsement, members who were acquainted with the applicants were requested to indorse them.

### PROPOSED PUBLICATION.

It was moved by Mr. Bartlette, that the Board of Trustees be, and are hereby, authorized to permit any person or persons who may desire to establish, in this city, a monthly journal, devoted to the interests of chemistry, pharmacy, and the collateral sciences, and who shall present in writing a full prospectus of the enterprise, and shall give satisfactory evidence of their qualifications and responsibility, to publish the same by authority of the Chicago College of Pharmacy, and the authority so delegated shall continue so long as the requirements of the prospectus are fulfilled, or until the College may desire such a journal. The motion was carried without debate.

It was moved and adopted, that the Board of Trustees be empowered to take measures to give a course of lectures on pharmacy.

On motion, the Secretary was empowered to transmit a vote of thanks to Dr. J. H. Rauch, for a valuable donation.

On motion, the unappropriated Library fund was placed in the hands of the Trustees.

It was also resolved, that $300 be placed in the hands of the Trustees, to defray the current expenses.

PRESIDENT'S ADDRESS.

President Sargent then addressed the meeting substantially as follows:

GENTLEMEN:—Another annual meeting brings us together, and it becomes my pleasing duty to present to you a statement of what has been done by your officers, which, owing to the change of our Constitution, covers a space of only six months.

He congratulated them upon the increased prosperity of the College, and the prospect of greater future usefulness. But twelve months had passed since the reorganization, and it was not much to say that the College is far more prosperous than the most sanguine could have anticipated. But there remained much to be done before they could rank with older and more favored Colleges.

It was to be regretted that so little had been done towards giving a course of instruction, but it was hoped that this meeting would take the matter under consideration, and take steps necessary to its fulfilment.

Our city was large enough to furnish a class of students sufficient to sustain a good course of lectures. There is not a druggist in Chicago whose clerks would not benefited by a course of lectures. For such lectures, but six hours a week would be required; and he thought that each clerk would be willing to devote a portion of his labor and leisure hours to study.

All desiring proper, competent clerks, would give preference to the graduates of the College of Pharmacy. Such clerks usually obtain a large salary.

The class in the Philadelphia College of Pharmacy numbers one hundred and fifty members, two-thirds of whom are from the city proper. Several Chicago clerks annually go to that College to obtain the advantages which cannot be found here.

The influence of Chicago will extend over the entire West. We may be sure that in a short time our classes will be as largely attended as those of any similar institution in the country.

We ought and can furnish the necessary talent to give the lectures; the only difficulty being to find the necessary time to write and deliver the lectures, but with suitable compensation the difficulty may be overcome.

From the Secretary we learn that six new members have united with the College since September, making the number 83.

The library now contains 125 volumes, comprising many of the best books in the language upon chemistry and collateral branches of sciences and art. The most complete and expensive books have been purchased rendering the library one of great utility.

In view of the great cost of scientific books, and the fact that their resources are limited, the speaker urged upon all to aid, by the donation of books and publications. to the Society. He also suggested that the Committee on Library issue a circular, addressed to public libraries, officers and individuals, asking contributions. Publishers and dealers might also be included.

The cabinet had not been neglected, it now comprised over

700 specimens. Each one should make a selection from his private stock of what is needed. It only required a moderate outlay of time and money on the part of each one to make the Cabinet what it should be—a means of instruction. He called attention to the importance of having specimens of adulterations, and asked that each donate such as might fall under their notice. He also suggested that the Committee should solicit from abroad what could not be obtained at home.

He took pleasure in informing them that Mr. Ebert, who is now in Europe, had a collection of over 600 specimens, and promises many more.

Attention of the members was called to the subject of a cabinet of chemical and pharmacal apparatus. Such a collection would be as valuable as any other portion of our cabinet.

In January last, a lease was secured of a building now occupied, on very favorable terms.

He hoped that during the ensuing year, the College would be able to invite the American Pharmaceutical Association to meet in Chicago. The Association has held fifteen annual meetings: four times in New York, three times in Boston, twice in Cincinnati, twice in Baltimore, twice in Philadelphia, where they will meet again this year. The membership of our State ranks as the sixth in number, Chicago containing all but six, having forty-three members. The largest number of any Western City, except Cincinnati, which has sixty-one members.

Our revenue for the year now commenced is estimated will be about $500: the expenses, $400, independent of books and lectures.

Appropriations for current expenses should be made at this Session, as no money can be expended by the Trustees unless appropriated by the College.

The propriety of admitting wholesale druggists as associate members has been urged. It is desirable that as many of the members as can will become life members at once.

In conclusion, he urged upon all renewed interest in the work. In retiring from the office of President, he thanked them for the uniform courtesy they had shown him, and hoped that they would choose some one who would perform more worthily the duties of President, and that they would support the new officer as heartily as they had done him.

At the conclusion of the President's address, which was greeted with considerable applause, an election of officers was had, with the following result:

*President*—E. H. SARGENT.

*1st Vice-President*—J. W. MILL.

*2d Vice-President*—J. W. EHRMAN.

*Treasurer*—J. P. SHARP.

*Secretary*—LOUIS STREHL.

*Corresponding Secretary*—THOMAS WHITFIELD.

*Trustees*—HENRY BIROTH, W. F. BLOCKI, H. D. GARRISON, ALBERT E. EBERT, and N. GRAY BARTLETT.

The meeting then adjourned.

